# Pararamosis, a Neglected Tropical Disease Induced by *Premolis semirufa* Caterpillar Toxins: Investigating Their Effects on Synovial Cell Inflammation

**DOI:** 10.3390/ijms252313149

**Published:** 2024-12-06

**Authors:** Paula C. Pohl, Isadora M. Villas-Boas, Giselle Pidde, Denise V. Tambourgi

**Affiliations:** Immunochemistry Laboratory, Instituto Butantan, São Paulo 05503-900, Brazil; paula.pohl@butantan.gov.br (P.C.P.); isadora.boas@butantan.gov.br (I.M.V.-B.); giselle.pidde@butantan.gov.br (G.P.)

**Keywords:** pararamosis, *Premolis semirufa*, coculture, synovial cell crosstalk, cartilage breakdown, inflammation

## Abstract

Pararamosis, also known as Pararama-associated phalangeal periarthritis, is a neglected tropical disease primarily affecting rubber tappers in the Amazon region. It is caused by contact with the urticating hairs of the *Premolis semirufa* moth caterpillar, which resides in rubber plantations. The condition is marked by the thickening of the articular synovial membrane and cartilage impairment, features associated with chronic synovitis. Given the significance of synovial inflammation in osteoarticular diseases, in this study, the role of synoviocytes and their interactions with macrophages and chondrocytes are examined when stimulated by Pararama toxins. Synoviocytes and macrophages treated with Pararama hair extract showed an increased production of cytokines IL-6, IL-1β, and TNF-α, indicating a direct effect on these cells. In cocultures, there was a significant rise in inflammation, with levels of IL-1β, IL-6, and chemokines CCL2, CCL5, and CXCL8 increasing up to seven times compared to monocultures. Additionally, matrix-degrading enzymes MMP-1 and MMP-3 were significantly elevated in cocultures. Chondrocytes exposed to the extract also produced IL-6, CCL2, and CCL5, and in cocultures with synoviocytes, there was a notable increase in IL-6, CCL5, and CXCL8, as well as a doubling of MMP-1 and MMP-3 levels. These findings underscore the critical role of cell crosstalk in the inflammatory and catabolic processes associated with pararamosis and demonstrate how Pararama hair extract can influence factors affecting cartilage health, providing valuable insights into this condition.

## 1. Introduction

Pararamosis, also known as Pararama-associated phalangeal periarthritis, is a neglected tropical disease primarily affecting rubber tappers in the Amazon region. This condition arises from contact with the urticating hairs of the caterpillar of the *Premolis semirufa* moth, commonly referred to as Pararama, which inhabits rubber plantations [1,2]. The disease poses significant health concerns due to its chronic inflammatory nature and detrimental effects on joint health. Initially, it manifests symptoms such as itching and acute inflammation, resembling other forms of erucism (injuries caused by moth larvae in humans). However, prolonged exposure to the hairs of the Pararama caterpillar can lead to severe reactions, including persistent inflammation, cartilage damage, and skeletal deformities [2,3]. X-rays of the affected hands from individuals envenomated by Pararama demonstrate swelling of the soft tissues, along with fibrosis of the juxta-articular tissue and loss of joint space due to thickening of the synovial membrane. Additionally, deformities of the interphalangeal joints can be observed in some patients, resembling the manifestations seen in other articular diseases, such as osteoarthritis (OA) [2,4,5].

Our research group is focused on elucidating the mechanisms underlying pararamosis to better assess its impact on joint health. We have previously demonstrated that extracts from Pararama hairs induce a robust inflammatory response in the joints of inoculated mice, marked by the infiltration of macrophages and neutrophils into the affected areas [6]. Further investigations have revealed that these extracts contain toxins capable of activating the human complement system, resulting in the production of anaphylatoxins such as C3a, C4a and C5a, which play a role in the inflammatory response [7,8]. Additionally, a serine protease with proteolytic activity found in the hair extract exacerbates tissue damage and inflammation [8]. Multi-omics analyses of *P. semirufa* caterpillar venom have identified homologous proteins to human proteins involved in critical biological processes, including chemotaxis, cell adhesion, apoptosis, cytoskeletal remodeling, and extracellular matrix (ECM) remodeling. These processes are essential in the context of joint diseases, underscoring the pathological similarities between pararamosis and other articular disorders [9].

Our studies have also demonstrated that human chondrocytes exposed to Pararama hair extract undergo significant changes, including an increased release of pro-inflammatory cytokines (such as IL-6 and IL-8), chemokines (like CCL2), prostaglandin E2, and components of the complement system. Additionally, we observed an increased production of metalloproteinases (MMP-1, MMP-2, MMP-3, and MMP-13), which are enzymes that degrade the ECM and contribute to cartilage destruction [10]. Transcriptomic analyses further corroborated these findings, revealing the activation of inflammatory pathways, immune cell chemotaxis, and ECM remodeling in chondrocytes treated with Pararama hair extract [10].

Although they arise from different causes, pararamosis and OA share several similarities, particularly in their inflammatory and degenerative characteristics. In OA, synovial inflammation (synovitis) plays a crucial role, with synoviocytes and macrophages being pivotal in the production of inflammatory mediators and degradative enzymes [11,12,13]. Early changes in the synovium, which occur before visible cartilage degeneration, are observed in OA. They include mononuclear cell infiltration, thickening of the synovial lining, and the release of inflammatory cytokines. A hallmark of synovitis is the accumulation of polarized macrophages in the intimal lining [14,15]. These macrophages, known for their plasticity, respond to damage-associated molecular patterns (DAMPs) by releasing inflammatory mediators such as IL-1β, TNF-α, and CCL2. This response recruits additional macrophages and stimulates the production of degradative enzymes by chondrocytes and synoviocytes, which cause the subsequent cartilage degradation [14,16,17,18].

These observations suggest a shared immune response pathway that exacerbates joint damage in both diseases; however, the significance of synoviocytes and their interactions with surrounding cells in pararamosis disease remains unclear. Therefore, in this study, the role of synoviocytes and their interactions with macrophages and chondrocytes in producing soluble inflammatory mediators and degradative enzymes in response to stimulation by *P. semirufa* caterpillar toxins are investigated. We discuss the implications of these interactions for the pathophysiology of pararamosis. Understanding these interactions can aid in recognizing the potential for similar treatment approaches for pararamosis and osteoarthritis, focusing on managing inflammation and promoting joint health.

## 2. Results

### 2.1. Human Synoviocytes Are Activated by Pararama Hair Extract

To evaluate the impact of Pararama hair extract on human synoviocytes, we assessed cell viability following exposure to three different concentrations of the extract (15, 30, and 60 µg/mL) over 24, 48, and 72 h. As shown in Supplementary Figure S1, there was a minimal decrease in synoviocyte viability with Pararama hair extract treatment, except at the highest concentration.

**Figure 1:**
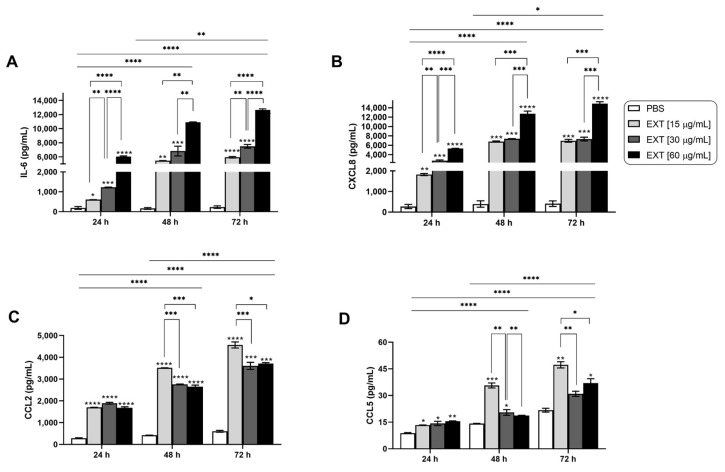
Cytokine and chemokine levels in synoviocyte cultures after Pararama hair extract stimulation. Synoviocytes (1 × 10^4^ cells/well) were treated with Pararama hair extract (EXT 15, 30, or 60 μg/mL) or PBS for 24, 48, or 72 h. After each treatment period, the supernatants were collected to assess the concentrations of cytokines and chemokines via a cytometric bead array (CBA) (**A**–**D**). The results represent two separate experiments performed in triplicate and are expressed as the mean of the concentrations of the molecules ± SEM. Statistical analyses within the same incubation time were performed using one-way ANOVA and Tukey’s post hoc test, while statistical analyses between different incubation times were conducted using two-way ANOVA and Tukey’s post hoc test. * *p* ≤ 0.05, ** *p* ≤ 0.01; *** *p* ≤ 0.001; and **** *p* ≤ 0.0001.

**Figure 2:**
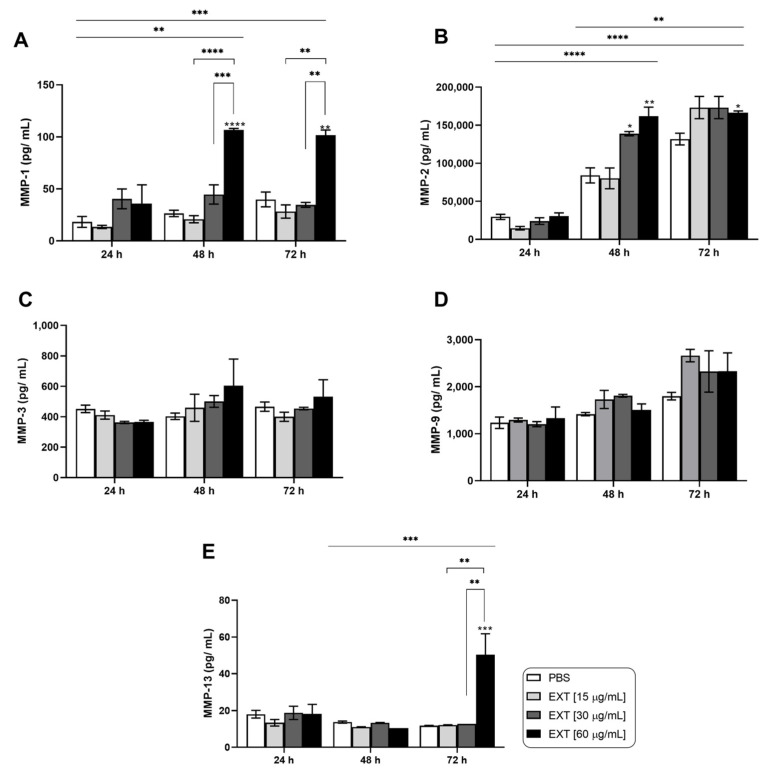
Metalloproteinase levels in synoviocyte culture after Pararama hair extract stimulation. Synoviocytes (1 × 10^4^ cells/well) were treated with Pararama hair extract (EXT 15, 30, or 60 μg/mL) or PBS for 24, 48, or 72 h. After stimulation, the production of metalloproteinases in the supernatants of the cultures was determined by enzyme-linked immunosorbent assay (ELISA) (**A**–**E**). The results represent two separate experiments performed in triplicate and are expressed as the mean of the concentrations of the molecules ± SEM. Statistical analyses within the same incubation time were performed using one-way ANOVA and Tukey’s post hoc test, while statistical analyses between different incubation times were conducted using two-way ANOVA and Tukey’s post hoc test. * *p* ≤ 0.05, ** *p* ≤ 0.01; *** *p* ≤ 0.001; and **** *p* ≤ 0.0001.

Given the critical role of inflammatory mediators and tissue-destructive enzymes in osteoarticular diseases, we measured the production of cytokines, chemokines, and metalloproteinases (MMPs) in the supernatants of synoviocytes treated with Pararama hair extract. Figure 1A,B illustrate that IL-6 and CXCL8 (IL-8) were significantly induced in a dose- and time-dependent manner in synoviocytes treated with the extract compared to those treated with the negative control (PBS). The chemokines CCL2 (MCP-1) and CCL5 (RANTES) were also released in response to Pararama hair extract stimulation in a time-dependent manner, with the lowest concentration of the extract inducing more pronounced effects (Figure 1C,D). Although IL-1β, IL-10, and TNF-α were quantified, they were not detected under any of the conditions tested. Analysis of MMP release revealed a significant increase in MMP-1 and MMP-2 only after 48 and 72 h of exposure to the highest concentration of Pararama hair extract (Figure 2A,B). Other MMPs did not show significant modulation in this assay (Figure 2C–E). These findings highlight the potential of Pararama hair extract to activate human synoviocytes, leading to an increased production of inflammatory mediators and enzymes that may contribute to the pathophysiology of pararamosis.

### 2.2. Proinflammatory Effects of Premolis semirufa Caterpillar Toxins on Macrophages and Synoviocytes in Coculture

To analyze the effects of soluble factors released by macrophages on human synoviocytes after stimulation with Pararama hair extract, a coculture system was established using transwell technology. First, we determined the efficiency of THP-1 cell differentiation into macrophages. Macrophages (MΦ) were differentiated from THP-1 cells seeded in the porous transwell insert. Following a resting period, these macrophages were activated to a pro-inflammatory M1 phenotype using lipopolysaccharide (LPS). After 48 h of treatment, the cytokines released in the cell supernatant were quantified, confirming successful macrophage activation, evidenced by a significant increase in the production of pro-inflammatory cytokines IL-1β, IL-6, and TNF-α compared to control MΦ macrophages that were not stimulated with LPS, which secreted minimal cytokines into the culture medium (Figure S2).

Subsequently, the extent of cell crosstalk between macrophages and synoviocytes in the presence of Pararama hair extract was analyzed within our established coculture system. A schematic representation of the workflow and treatment conditions is provided in Supplementary Figure S3A. Notably, only a slight reduction in the viability of both macrophages and synoviocytes was observed following treatment with Pararama hair extract under cocultivation conditions (Supplementary Figure S3B).

**Figure 3:**
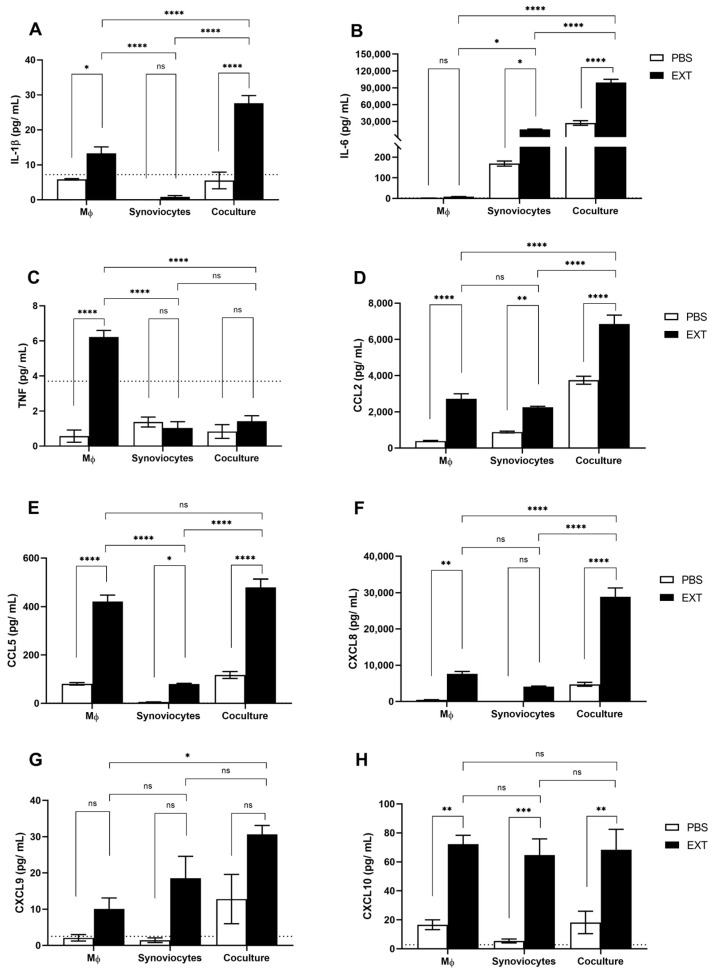
Cytokine and chemokine levels in cocultured synoviocytes and macrophages induced by Pararama hair extract. THP1-derived macrophages (MΦs), synoviocytes, and cocultured cells in the transwell system were treated with Pararama hair extract (EXT- 48 µg/mL) or PBS (as a negative control) for 48 h. Supernatants of the cultures were collected to assess the concentrations of cytokines and chemokines via a cytometric bead array (CBA) (**A**–**H**). The results summarize two independent experiments, each performed in triplicate, and are expressed as the means of the concentrations of the molecules ± SEM. The data were analyzed using two-way ANOVA and Tukey’s post hoc test. ns = not significant; * *p* ≤ 0.05, ** *p* ≤ 0.01, *** *p* ≤ 0.001, and **** *p* ≤ 0.0001. The dotted line represents the limit of detection for each analyte.

After 48 h of treatment with Pararama hair extract, we quantified the concentrations of cytokines and chemokines released in both the mono- and coculture supernatants. In the presence of Pararama hair extract, THP-1-derived macrophage monocultures produced low but statistically significant amounts of IL-1β and TNF-α compared to the negative control (PBS) (Figure 3A,C). Although IL-10 was also measured, it was not detected under any experimental conditions. These findings indicate that Pararama hair extract stimulates the release of inflammatory cytokines from macrophages, suggesting its potential to activate and polarize critical immune cells, such as macrophages, within the synovium.

The most important inflammatory mediators in OA are IL-1β, TNF-α, and IL-6. These cytokines act as inducers of various signaling pathways, which subsequently activate other pathological processes in joint cells, contributing to the progression of the disease. In experiments with synoviocyte monocultures, significant amounts of IL-6 were produced upon treatment with Pararama hair extract (Figure 3B), while the levels of TNF-α and IL-1β remained unchanged (Figure 3A,C). Notably, the concentrations of IL-1β and IL-6 in the supernatant of cocultured cells treated with Pararama hair extract were markedly higher than those produced by monocultures under identical conditions (Figure 3A,B). This increase in cytokine levels was not accompanied by TNF-α production, which was significantly lower, suggesting potential consumption of this cytokine by the cells (Figure 3C).

The chemokines CCL2, CCL5, and CXCL8, known for their strong monocytic chemo-attractive properties and correlation with OA severity, were significantly produced by macrophages and synoviocytes in monocultures following treatment with Pararama hair extract. In cocultured cells, the production of CCL2 and CXCL8 was significantly enhanced, whereas CCL5 production was comparable to that observed in macrophage monocultures but greater than in synoviocyte monocultures after stimulation (Figure 3D–F). Conversely, CXCL9 showed no significant modulation (Figure 3G), while CXCL10, another chemokine associated with OA severity, exhibited significant alterations in both monocultured and cocultured cells after treatment; however, no significant differences were noted between the culture types (Figure 3H).

**Figure 4:**
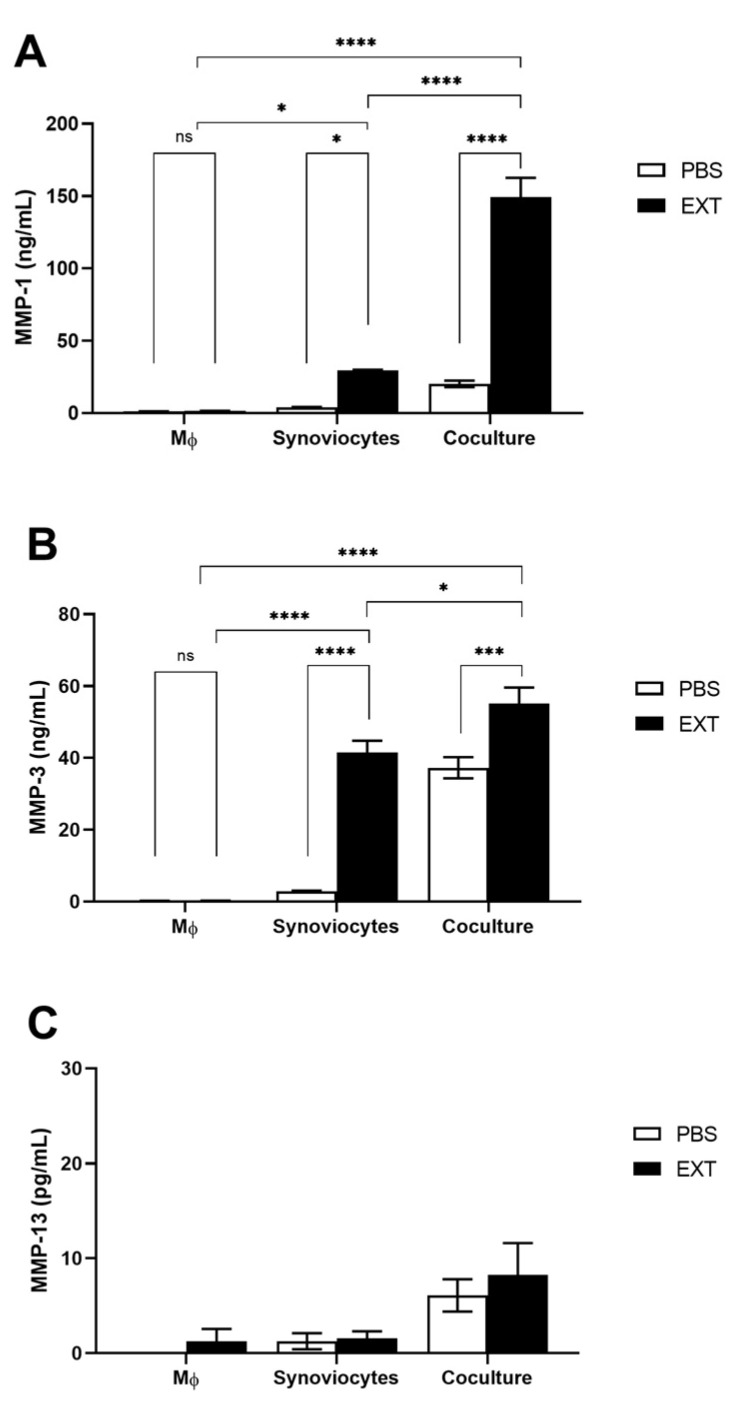
Metalloproteinase levels in cocultured synoviocytes and macrophages induced by Pararama hair extract. THP1-derived macrophages (MΦ), synoviocytes, and cocultured cells in the transwell system were treated with Pararama hair extract (EXT- 48 µg/mL) or PBS (as a negative control) for 48 h. Supernatants of the cultures were collected to assess the concentration of metalloproteinases by enzyme-linked immunosorbent assay (ELISA) (**A**–**C**). The results summarize two independent experiments, each performed in triplicate, and are expressed as the mean of the concentrations of the molecules ± SEM. The data were analyzed using two-way ANOVA and Tukey’s post hoc test. ns = not significant; * *p* ≤ 0.05, *** *p* ≤ 0.001, and **** *p* ≤ 0.0001. The dotted line represents the limit of detection for each analyte.

Given that activated synoviocytes in the inflamed OA synovium are stimulated to produce proteolytic enzymes that contribute to cartilage degradation, we assessed the release of MMPs in the supernatants of both mono- and cocultures. While stimulated macrophages did not produce MMPs, the levels of MMP-1 and MMP-3 were significantly elevated in synoviocyte monocultures treated with Pararama hair extract. Interestingly, the release of these MMPs was significantly greater in cocultured cells compared to monocultured cells under the same stimulus (Figure 4A,B), highlighting the crucial role of cell communication in exacerbating the pathological process. However, the expression of MMP-13, a key marker for OA, remained unchanged in this coculture system (Figure 4C).

### 2.3. Crosstalk Between Synoviocytes and Chondrocytes Produces Additional Cytokines and Proteolytic Enzymes

To analyze the effects of soluble factors released into the medium by synoviocytes on chondrocytes after Pararama hair extract treatment, the same transwell coculture system was used. A schematic representation of the workflow and treatment conditions is presented in Supplementary Figure S4A. Cell viability analysis revealed a small reduction in the viability of synoviocytes under monoculture or coculture conditions, while chondrocytes were slightly more sensitive to Pararama hair extract treatment (Supplementary Figure S4B).

**Figure 5:**
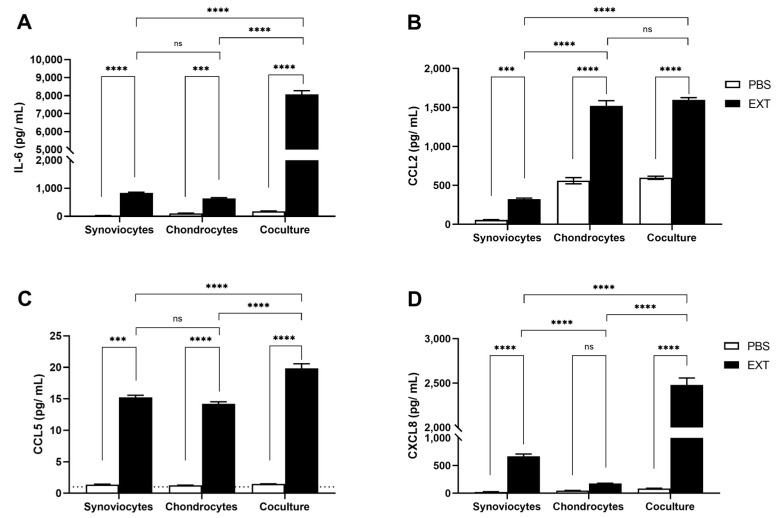
Cytokine and chemokine levels in cocultured synoviocytes and chondrocytes induced by Pararama hair extract. Synoviocytes, chondrocytes, and cocultured cells in the transwell system were treated with Pararama hair extract (EXT; 48 µg/mL) or PBS (as a negative control) for 48 h. Supernatants of the cultures were collected to assess the concentrations of cytokines and chemokines via a cytometric bead array (CBA) (**A**–**D**). The results summarize two independent experiments, each performed in triplicate, and are expressed as the means of the concentrations of the molecules ± SEM. The data were analyzed using two-way ANOVA and Tukey’s post hoc test. ns = not significant; *** *p* ≤ 0.001, and **** *p* ≤ 0.0001. The dotted line represents the limit of detection for each analyte.

After stimulation with Pararama hair extract, chondrocyte monocultures produced significant amounts of IL-6, CCL2, and CCL5, but CXCL8 was not significantly produced by chondrocytes in this study. Surprisingly, significantly greater amounts of IL-6 and CXCL8 were produced by chondrocytes and synoviocytes under coculture conditions. Similarly, CCL5 was detected at higher concentrations in coculture, while the CCL2 concentration after Pararama hair extract treatment was similar in both coculture and chondrocyte monoculture (Figure 5). The inflammatory mediators IL-1β, IL-10, TNF-α, CXCL9, and CXCL10 were not detected in the cell supernatants of the monocultures nor in the coculture of synoviocytes and chondrocytes (Figure S5).

**Figure 6:**
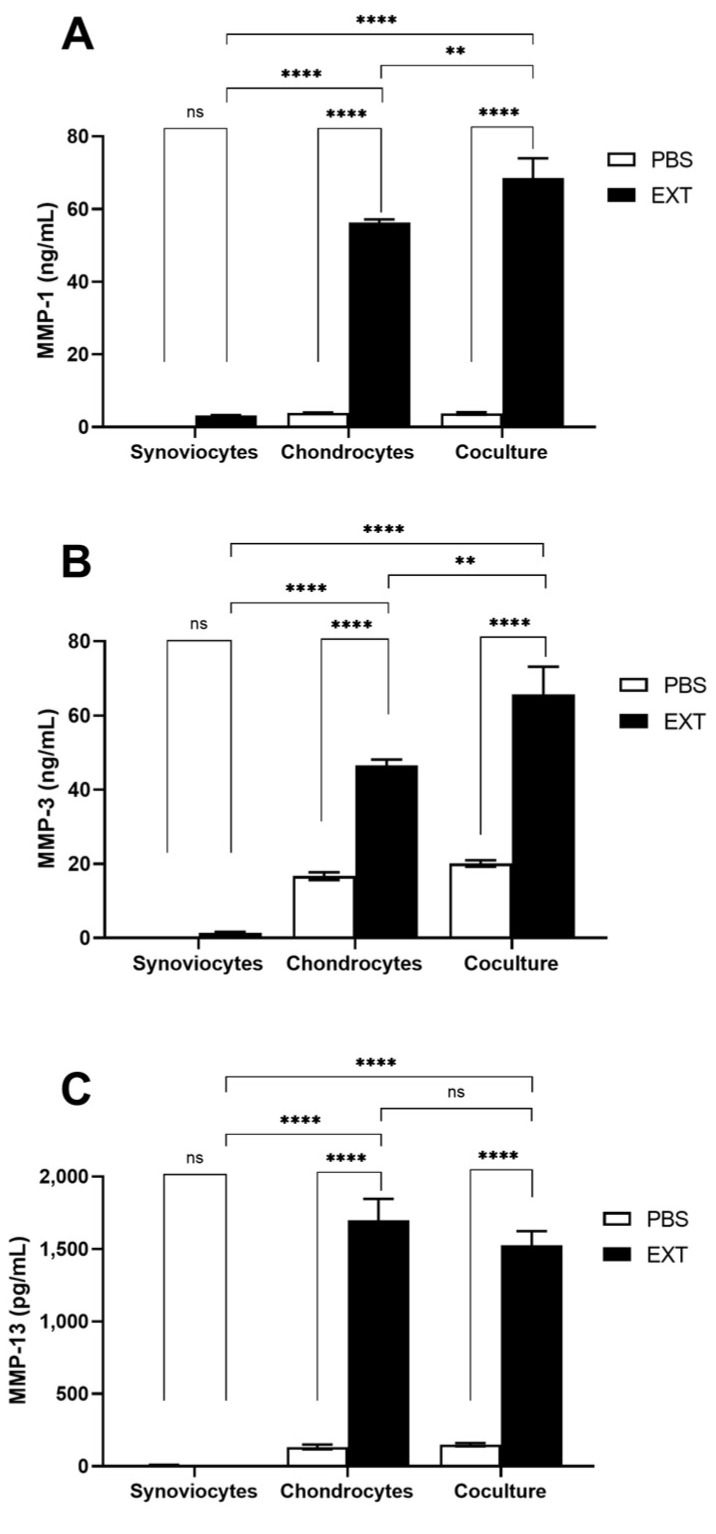
Metalloproteinase levels in synoviocyte and chondrocyte cocultures induced by Pararama hair extract. Synoviocytes, chondrocytes, and cocultured cells in the transwell system were treated with Pararama hair extract (EXT- 48 µg/mL) or PBS (as a negative control) for 48 h. Supernatants of the cultures were collected to assess the concentration of metalloproteinases by enzyme-linked immunosorbent assay (ELISA) (**A**–**C**). The results summarize two independent experiments, each performed in triplicate, and are expressed as the means of the concentrations of the molecules ± SEM. The data were analyzed using two-way ANOVA and Tukey’s post hoc test. ns-not significant, ** *p* ≤ 0.01; and **** *p* ≤ 0.0001.

We also evaluated the release of MMPs in the supernatants of both mono- and cocultures. The production of MMP-1 and MMP-3 was stimulated in chondrocyte monocultures following treatment with Pararama hair extract and was significantly increased in cocultured cells compared to monocultures (Figure 6A,B). Interestingly, while MMP-13 production was significantly elevated in chondrocyte monocultures after stimulation with Pararama hair extract, the levels in cocultures remained similar to those in monocultures (Figure 6C). These results highlight the critical role of chondrocytes in pararamosis and the influence of cell interactions on MMPs production, which may contribute to the inflammatory response and tissue degradation in the joint.

## 3. Discussion

Occupational joint disease, known as pararamosis, is a neglected tropical disease mainly affecting rubber tappers in the Amazonian region [1,2]. The inflammatory reactions observed in pararamosis have been studied by our group for more than one decade [6,7,8,9,10], and a large amount of evidence has shown its similarity with OA, the most prevalent joint disorder in the world [19]. The similarities between OA and pararamosis regarding the production of inflammatory factors are summarized in Supplementary Table S1.

Pharmacological treatments for both pararamosis and OA primarily focus on symptom relief rather than curing the conditions. For pararamosis, oral antihistamines are recommended to control itching, while topical corticosteroids and pain relievers are suggested to alleviate symptoms in the early stages. Unfortunately, no specific therapy is currently available for patients with chronic pararamosis [2]. In the case of OA, patients have access to topical, oral, and injectable pharmacological therapies aimed at reducing pain and discomfort. However, these therapies are generally only moderately effective, with first-line treatments including topical NSAIDs and oral paracetamol. Additionally, there are no disease-modifying drugs—treatments that reduce symptoms while also slowing or stopping disease progression—currently approved by regulatory agencies [19,20]. Exploring the potential synergies between the management of both diseases could lead to novel approaches for treating Pararamosis.

As a whole-organ disease, OA affects not only cartilage but also the surrounding tissues, and the influence of different joint compartments on OA pathogenesis has been demonstrated [21,22]. Synoviocytes are important cells in OA pathologies. They react to matrix fragments by producing pro-inflammatory mediators, which in turn attract immune cells, increase angiogenesis, and induce a phenotypic shift in chondrocytes [23,24].

In this study, we aimed to assess the effects of Pararama hair extract on synoviocytes by characterizing its interactions with the two key cell types involved in the onset of joint diseases, macrophages and chondrocytes.

The analysis of cytokines and chemokines in the supernatant of monocultured synoviocytes treated with Pararama hair extract revealed a dose- and/or time-dependent increase in the levels of IL-6, CXCL8, CCL5, and CCL2. This finding indicates a direct effect of the extract’s components on these cells (Figure 1).

To investigate the effects of soluble factors released by macrophages on synoviocytes following stimulation with Pararama hair extract, we established a transwell coculture system. Monocultured macrophages were stimulated to produce key inflammatory cytokines, including IL-1β and TNF-α, as well as the chemokines CCL2, CCL5, and CXCL8 (Figure 3). These results suggest that Pararama hair extract has the potential to activate and polarize macrophages. Additionally, our findings demonstrated that the incubation of synoviocytes with macrophages resulted in a significant increase in the concentration of inflammatory mediators compared to the monocultured cells, as follows: CCL2 increased threefold, CCL5 sixfold, and IL-6 also showed a sixfold increase, while CXCL8 rose by more than sevenfold. Interestingly, the concentration of IL-1β, which is not released by monocultured synoviocytes, increased twofold in the coculture supernatant compared to that in the monoculture of macrophages under the same stimulus (Figure 3). These results suggest that Pararama hair extract has pro-inflammatory effects and highlight the crosstalk between these cells, which exacerbates the inflammatory response. Indeed, radiographic OA severity and related joint symptoms have been shown to correlate with the number of activated macrophages in the synovial tissue of individuals with knee OA [25]. Similarly, our previous report demonstrated the infiltration of macrophages into the joints of mice inoculated with Pararama hair extract [6], reinforcing the importance of macrophage interactions with synoviocytes.

Comparably, the key inflammatory mediators in OA include IL-1β, TNF-α, and IL-6, which are present at elevated concentrations in the plasma, synovial fluid, and articular cartilage of OA patients compared to healthy controls [26,27,28]. These cytokines activate a variety of signaling pathways that trigger the production of other cytokines and contribute to pathological processes. When stimulated by these cytokines, the production of chemokines increases, attracting more inflammatory cells to the joint and further promoting the secretion of inflammatory factors [27]. Among the most significant chemokines associated with OA are CCL2, CCL5, and CXCL8 [27,29,30]. CCL2, also known as MCP-1, is a potent chemotactic factor for monocytes in joint tissues [31,32]. Elevated levels of CCL2 have been found in the synovial fluid of patients with both knee injuries and knee OA [28], and its levels correlate positively with pain and physical disability in these patients [29]. CCL5 (or RANTES) is one of the most significantly elevated mediators in the synovial fluid of OA patients compared to controls [26]. CCL5 primarily attracts lymphocytes, monocytes, and other cell types. While CCL5 induces matrix metalloproteinase (MMP) production in rheumatoid arthritis, its role in OA is not yet well defined [32,33]. CXCL8 is also upregulated in OA tissues [27,28]; it is a potent mediator of inflammation and angiogenesis, affecting various cell types, including chondrocytes, osteoclasts, fibroblasts, and both epithelial and endothelial cells [34].

Although information regarding inflammatory mediators in the plasma or synovial fluid of pararamosis patients is currently lacking, studies have investigated inflammatory responses in animal models. In our previous study, BALB/c mice were subcutaneously injected in the footpad with Pararama hair extract, resulting in increased levels of innate immunity-associated cytokines such as TNF-α and IL-6. Interestingly, the production of IL-1 remained unchanged. These findings align with our observations; however, we noted a small but consistent increase in IL-1β.

Chronic inflammation in joint diseases can also be driven by modified proteins and subsequent immune reactions, as seen with methylglyoxal-modified IgG in rheumatoid arthritis (RA), a chronic autoimmune disease that primarily affects the joints [35]. Despite the similarities between pararamosis and RA regarding certain inflammatory features, the disease induced by contact with *Premolis semirufa* does not appear to be a systemic autoimmune disorder, as it does not lead to the generation of autoantibodies such as anti-DNA or anti-collagen type II, as reported by Villas-Boas et al. [3].

We also evaluated the release of MMPs in the supernatants of both mono- and cocultures. MMPs play crucial roles in tissue differentiation, healing, organ formation, reproduction, blood vessel formation, and tissue remodeling. However, they are also highly expressed in pathological conditions such as OA, where they significantly contribute to disease pathogenesis [36]. In the presence of Pararama hair extract, monocultured synoviocytes produced MMP-1 and MMP-3, while these MMPs were not detected in macrophage monocultures. Notably, in cocultures, the levels of MMP-1 and MMP-3 in the supernatant increased by 5-fold and 1.5-fold, respectively (Figure 4).

Although some discrepancies were observed in the modulation of MMPs and cytokines between the different experimental conditions to which monocultured synoviocytes were subjected, these may reflect variations in the cell numbers present in each condition (1 × 10^4^ cells/well or 1 × 10^5^ cells/well), which can affect the levels of MMPs produced. Additionally, limitations in the sensitivity of the quantification assays may also contribute to the lack of detection for certain cytokines.

A recent study by Chou et al. [22] was the first to explore the molecular crosstalk between cartilage and the synovium in OA using single-cell RNA sequencing. They evaluated the potential origins of upstream cytokines that may drive phenotypic changes in OA chondrocytes. Their findings indicated that key inflammatory mediators in OA, including TNF-α, IL-1β, and IL-6, are predominantly produced and released into the joint space by synovial cells, particularly macrophages, dendritic cells, and synoviocytes. IL-1β and TNF-α act through similar signaling pathways, resulting in synergistic effects that promote catabolic events in chondrocytes, such as cartilage degradation, which is the predominant process in OA. They facilitate ECM degradation by inducing the expression of collagenases and aggrecanases, including MMP-1, MMP-3, MMP-13, ADAMTS-4, and ADAMTS-5 [27,37]. Through this signaling pathway, IL-1β also stimulates the secretion of IL-6 and promotes its own upregulation [38]. Additionally, the level of IL-6, a pleiotropic cytokine with both pro- and anti-inflammatory properties, is significantly elevated in the synovial fluid of OA patients [26]. Its concentration correlates with pain and increased loss of cartilage volume [39]. IL-6 signals through the PI3K, JAK/STAT, and MAPK pathways, regulating the production of enzymes (including TIMP, MMPs, and ADAMTS) as well as the synthesis of type II collagen and proteoglycans [40]. Thus, while IL-6 balances anti-inflammatory and pro-inflammatory effects, the latter predominates, ultimately contributing to the progression of the osteoarticular disease.

Exacerbated inflammation in the synovial membrane significantly influences chondrocyte metabolism, leading to hypertrophy, dedifferentiation, and ultimately, apoptosis [41]. To investigate this further, we evaluated the crosstalk between synoviocytes and chondrocytes in the presence of Pararama hair extract. As previously reported, Pararama hair extract alters the phenotype of chondrocytes, inducing inflammation and extracellular matrix (ECM) remodeling [10]. Here, we observed that the levels of inflammatory mediators in coculture, particularly IL-6 and CXCL8, were more than 10-fold greater than those in monocultured chondrocytes (Figure 5). This heightened inflammatory response resulted in a doubling of MMP-1 and MMP-3 production. Interestingly, while MMP-13–an important marker of OA and the primary enzyme responsible for cartilage degradation [42,43]–was not modulated in the synoviocyte and macrophage coculture system, it was significantly produced by chondrocytes under the inflammatory stimulus induced by Pararama hair extract. However, no additive effect was observed in cocultured cells (Figure 6).

The toxic products produced by *P. semirufa* exhibit high hyaluronidase and metalloprotease activity [3,8], leading to the breakdown of hyaluronic acid and collagen, which are essential for joint stability, lubrication, and cartilage remodeling. In chondrocytes treated with Pararama hair extract, a notable reduction in collagen II—vital for healthy cartilage—was observed. The breakdown of ECM components activates signaling pathways such as NF-κB, triggering inflammatory cytokines and further activating JAK/STAT and MAPK pathways, which increase MMP production, as shown in transcriptome analyses [10]. Corroborating this, in the present study, it was demonstrated that Pararama hair extract strongly activates synoviocytes, chondrocytes, and macrophages in coculture systems, intensifying the inflammatory response through cellular interactions that contribute to joint inflammation and degradation.

Coculture systems have been utilized for drug development, with most models based on synovial and cartilage tissues or cells [44,45,46]. Although in vivo studies are commonly performed to confirm the relevance of these interactions, there has been a growing interest in using in vitro coculture models as alternatives to reduce animal use in joint research [47,48]. However, it is important to acknowledge that while coculture systems provide valuable insights into cellular interactions and inflammatory responses, they may not fully replicate the complex physiological conditions present in vivo. Therefore, findings from these models should be interpreted with caution. In this context, coculture cell models induced by toxins from *P. semirufa* caterpillars could serve as a useful alternative for accurately evaluating the role of new anti-inflammatory or chondroprotective molecules for managing Pararamosis disease.

## 4. Materials and Methods

### 4.1. Pararama Hair Extract

Caterpillars of *P. semirufa* were gathered in São Francisco, Pará, Brazil, under licenses for capture and maintenance issued by the Brazilian Institute of Environment and Renewable Natural Resources (IBAMA), Brazil, with license number 45166–6. The caterpillar hairs were cut and placed in tubes containing phosphate-buffered saline (PBS) (8.1 mM Na_2_HPO_4_; 1.5 mM KH_2_PO_4_; 137 mM NaCl; 2.7 mM KCl; pH 7.4) before being frozen at −80 °C until required. For extract preparation, the samples were macerated using a glass rod, and centrifuged at 560× *g* for 20 min at 4 °C to remove insoluble material. The resulting supernatant was filtered through a 0.22 µm membrane (Whatman-GE Healthcare, Chicago, IL, USA), aliquoted, and stored at −80 °C. Protein concentration was determined using a bicinchoninic acid (BCA) protein assay kit (Pierce Biotechnology, Rockford, IL, USA) following the manufacturer’s instructions. Access to the venom was authorized by the National System of Genetic Resource Management and Associated Traditional Knowledge (SisGen) under registration number AEA2993.

### 4.2. Synoviocyte Monoculture

Human synoviocytes, from normal donors, were obtained from Articular Engineering (Chicago, IL, USA). Culturing and subculturing were carried out using Dulbecco’s modified Eagle’s medium/Nutrient F-12 Ham growth medium (DMEM/F12, Sigma-Aldrich, St. Louis, MO, USA) supplemented with 14 mM sodium bicarbonate, 1% penicillin-streptomycin solution (Gibco, Invitrogen Corp., Carlsbad, CA, USA), and 10% fetal bovine serum (FBS, Cultilab, São Paulo, SP, Brazil) in a humidified incubator at 37 °C and 5% CO_2_. For experimental purposes, cells at the 6th passage were seeded into 96-well plates (Costar^®^, Corning Inc., New York, NY, USA) at a density of 1 × 10^4^ cells/well and allowed to adhere overnight in DMEM/F12 supplemented with 10% FBS. Following attachment, cells were exposed to increasing concentrations of Pararama hair extract (15, 30, and 60 µg/mL) for 24, 48, and 72 h in DMEM/F12 supplemented with 1% FBS. As a negative control, synoviocytes were cultured in DMEM/F12 supplemented with 1% FBS and PBS, using the same volume as the extract. At the conclusion of each time interval, the supernatant was carefully removed, centrifuged at 405× *g* for 10 min at 4 °C to eliminate nonadherent cells, aliquoted, and stored at −80 °C for subsequent analysis. The proliferation and viability of the adhered cells were assessed using the MTT method.

### 4.3. THP-1-Derived Macrophage and Synoviocyte Coculture in a Transwell System

THP-1 cells, a human leukemia-derived monocytic cell line, were acquired from BCRJ (Rio de Janeiro, Brazil). The cells were cultured at a density of 0.2–1.0 × 10^6^ cells/mL in RPMI medium (Gibco, Invitrogen Corp., Carlsbad, CA, USA) enriched with 25 mM glucose, 10 mM HEPES, 1 mM sodium pyruvate, 24 mM sodium bicarbonate, 1% penicillin-streptomycin solution, and 10% FBS at 37 °C and 5% CO_2_. For coculture experiments, a transwell system was established. Three days before the stimulus, THP-1 cells were seeded at a density of 2.5 × 10^4^ cells/well into 96-well transwell inserts with 0.4 μM pore size (Costar^®^, Corning Inc., New York, NY, USA) and activated to macrophages (MΦs) with 50 nM phorbol 12-myristate 13-acetate (PMA, Sigma-Aldrich, St. Louis, MO, USA) for 48 h. Following activation, PMA-containing medium was removed, and the cells were incubated in fresh RPMI medium for 24 h. Simultaneously, 24 h before the stimulus, synoviocytes were seeded at a density of 1 × 10^5^ cells/well in 24-well plates (Costar^®^, Corning Inc., New York, NY, USA) and incubated for 24 h.

To initiate coculture, half of the inserts containing PMA-differentiated MΦs were transferred onto the top of the 24-well plates containing synoviocytes, while the other half of the wells remained isolated for comparative analysis as monoculture controls. Pararama hair extract (48 µg/mL) or PBS (the same volume as the extract) were added to the cell cultures in DMEM/F12 containing 1% FBS, in a total volume of 700 µL. After 48 h of incubation, the supernatant was collected, processed, and frozen for subsequent analysis. Cell proliferation and viability were evaluated using the MTT method (for more details, see Supplementary Figure S3A).

### 4.4. Response to Lipopolysaccharides

To validate the cell response, PMA-differentiated macrophages (MΦs) were stimulated with LPS (Escherichia coli strain O111:B4, Sigma-Aldrich, St. Louis, MO, USA) as a control. The stimulation was carried out for 48 h at a final concentration of 100 ng/mL. Subsequently, cytokine secretion was quantified in the cell culture supernatants, providing a benchmark for the cellular response to a known stimulus.

### 4.5. Coculture of Chondrocytes and Synoviocytes in a Transwell System

Human chondrocytes, specifically cryopreserved chondrocytes from normal donors, were obtained from Lonza (Walkersville, MD, USA). The cells were cultured up to the 3rd passage in chondrogenic growth medium supplemented with growth factors (Lonza, Walkersville, MD, USA) at 37 °C and 5% CO_2_. Subsequently, the medium was gradually replaced with DMEM/F12 medium (Sigma-Aldrich, St. Louis, MO, USA) supplemented with 14 mM sodium bicarbonate, 1% penicillin–streptomycin solution, and 10% FBS, reaching full transition by the 5th passage.

For coculture experiments, synoviocytes were seeded at a density of 1 × 10^4^ cells/well into 96-well transwell inserts. Simultaneously, chondrocytes at the 5th passage were seeded at a density of 1 × 10^5^ cells/well in 24-well plates and incubated for 24 h. To establish coculture, half of the inserts containing synoviocytes were placed on top of the 24-well plates with chondrocytes, while the remaining wells served as monoculture controls for comparative analysis. Subsequently, Pararama hair extract (48 µg/mL) or PBS (equivalent volume to the extract) were introduced to the cell cultures in DMEM/F12 supplemented with 1% FBS, totaling 700 µL. After 48 h of incubation, the supernatant was harvested, processed, and stored for subsequent analysis. Concurrently, cell proliferation and viability were assessed using the MTT method (for more details, see Supplementary Figure S4A).

### 4.6. Cell Viability Evaluation Using the MTT Method

The proliferation/viability of the adhered cells was evaluated by the MTT assay, which quantifies the levels of mitochondrial activity as a readout of cell viability. The assay is based on the absorption of MTT salt (3-(4,5-dimethylthiazol-2-yl)-2,5-diphenyltetrazolium bromide) (Sigma-Aldrich, St. Louis, MO, USA) by viable cells. Briefly, the cells were incubated with 0.5 mg/mL MTT in a volume of 100 µL of DMEM/F12 for 3 h at 37 °C and 5% CO_2_. During this period, MTT is incorporated into viable cells (metabolically active cells) and reduced with the formation of formazan crystals that accumulate in the cytoplasm. After dissolving the crystals in 100 µL of dimethyl sulfoxide (DMSO) (Merck, Darmstadt, Germany) for 10 min, the absorbance of the solution was determined at a wavelength (λ) of 540 nm using a spectrophotometer (FLUOstar Omega, BMG Labtech Inc., Durham, NC, USA).

### 4.7. Quantification of Cytokines and Chemokines

The concentrations of cytokines and chemokines in the supernatants of the cultures were analyzed by flow cytometry using the following BD Biosciences kits: BDTM Cytometric Bead Array (CBA) Human Inflammatory Cytokines and BDTM Cytometric Bead Array (CBA) Human Chemokine. The assays were performed according to the manufacturer’s recommendations (BD Biosciences, San Jose, CA, USA). The samples were evaluated for the presence of the cytokines IL-1β, IL-6, IL-10, IL-12p70, and TNF-α and for the chemokines CCL2/MCP-1, CCL5/RANTES, CXCL8/IL-8, CXCL9/MIG, and CXCL10/IP-10. The concentration of each factor was determined using FCAP Array 3.0 software (BD Biosciences, San Jose, CA, USA).

### 4.8. Evaluation of Matrix Metalloproteinases (MMPs)

The concentrations of MMP-1, MMP-2, MMP-3, MMP-9, and MMP-13 in the culture supernatants were assessed using commercially available human enzyme-linked immunosorbent assays (ELISAs) according to the manufacturer’s recommendations (Abcam, Cambridge, UK). For the analysis, a standard curve was built on the log-log graph for each MMP concentration using the software GraphPad Prism, version 10.3.1.

### 4.9. Statistical Analysis

The statistical significance of the results was determined using GraphPad Prism software, version 10.3.1. The analysis involved one-way ANOVA, two-way ANOVA, followed by Tukey’s post hoc test for multiple comparisons, or *t*-test. The results were considered statistically significant when the *p*-values met the following criteria: * *p* ≤ 0.05, ** *p* ≤ 0.01, *** *p* ≤ 0.001, and **** *p* ≤ 0.0001.

## 5. Conclusions and Future Perspectives

In summary, in this study, we provide essential insights into the inflammatory mechanisms linked to pararamosis, a neglected tropical disease resembling osteoarthritis. The findings reveal that Pararama hair extract notably stimulates synoviocytes, chondrocytes, and macrophages, resulting in the heightened production of pro-inflammatory mediators in coculture systems. This interplay among the cell types intensifies the inflammatory response, underscoring the complex interactions that lead to joint inflammation and degradation. These results emphasize the need for a deeper understanding of cellular interactions in joint diseases and suggest potential therapeutic avenues for addressing inflammation and cartilage degradation associated with pararamosis and similar osteoarticular disorders.

Looking ahead, future research will focus on assessing the effects of Pararama hair extract in relevant animal models to clarify the mechanisms driving the inflammatory response. Additionally, we plan to explore the efficacy of specific inhibitors in modulating the inflammatory pathways activated by these toxins. Such investigations aim to enhance our understanding of the pathophysiology of pararamosis and aid in identifying potential therapeutic targets for managing this inflammatory joint disease.

## Data Availability

The authors declare that all data supporting the findings of this study are available within the paper in the main text or the Supplementary Materials. Further inquiries can be directed at the corresponding author.

## Supplementary Materials

The following supporting information can be downloaded at: https://www.mdpi.com/article/10.3390/ijms252313149/s1 [49].

## References

[1] Costa R.M., Silva N.P., Leser P.G., Andrade L.E.C., Gabriel Junior A. (1995). Activity of Bristles from an Amazonian Lepidoptera, “*Premolis semirufa*”, on the Human Complement System. Rev. Bras. Reumatol..

[2] Siqueira-Batista R., Montenegro S.S.P., Novelli M.M., Feio R.N. (2021). Pararamosis: Disease of the Rubber Plantations. Am. J. Trop. Med. Hyg..

[3] Villas-Boas I.M., Gonçalves-de-Andrade R.M., Pidde-Queiroz G., Assaf S.L.M.R., Portaro F.C.V., Sant’Anna O.A., van den Berg C.W., Tambourgi D.V. (2012). *Premolis semirufa* (Walker, 1856) Envenomation, Disease Affecting Rubber Tappers of the Amazon: Searching for Caterpillar-Bristles Toxic Components. PLoS Negl. Trop. Dis..

[4] Costa R.M., Atra E., Ferraz M.B., Silva N.P., Souza J.M., Alves JR J., Costa M.L.C. (1993). “Pararamose”: An occupational arthritis caused by lepidoptera (*Premolis semirufa*). An epidemiological study. Rev. Paul. De Med..

[5] Barcelos A., Nour D., Silva J.A.P. (2001). Radiografia das mãos: Elementos típicos em artropatias comuns. Acta Reum. Port..

[6] Villas-Boas I.M., Gonçalves-de-Andrade R.M., Squaiella-Baptistão C.C., Sant’Anna O.A., Tambourgi D.V. (2013). Characterization of Phenotypes of Immune Cells and Cytokines Associated with Chronic Exposure to *Premolis semirufa* Caterpillar Bristles Extract. PLoS ONE.

[7] Gabrili J.J.M., Villas-Boas I.M., Pidde G., Squaiella-Baptistão C.C., Woodruff T.M., Tambourgi D.V. (2022). Complement System Inhibition Modulates the Inflammation Induced by the Venom of *Premolis semirufa*, an Amazon Rainforest Moth Caterpillar. Int. J. Mol. Sci..

[8] Villas-Boas I.M., Pidde-Queiroz G., Magnoli F.C., Gonçalves-de-Andrade R.M., Van Den Berg C.W., Tambourgi D.V. (2015). A Serine Protease Isolated from the Bristles of the Amazonic Caterpillar, *Premolis semirufa*, Is a Potent Complement System Activator. PLoS ONE.

[9] Pidde G., Nishiyama M.Y., de Oliveira U.C., Villas-Boas I.M., Paes-Leme A.F., Junqueira-de-Azevedo I.L., Marques-Porto R., Squaiella-Baptistão C.C., Tambourgi D.V. (2021). Integrative Multiomics Analysis of *Premolis semirufa* Caterpillar Venom in the Search for Molecules Leading to a Joint Disease. Sci. Rep..

[10] Villas-Boas I.M., Pidde G., Lichtenstein F., Ching A.T.C., Junqueira-de-Azevedo I.d.L.M., DeOcesano-Pereira C., Madureira Trufen C.E., Chudzinski-Tavassi A.M., Morais K.L.P., Tambourgi D.V. (2020). Human Chondrocyte Activation by Toxins From *Premolis semirufa*, an Amazon Rainforest Moth Caterpillar: Identifying an Osteoarthritis Signature. Front. Immunol..

[11] Hügle T., Geurts J. (2016). What Drives Osteoarthritis?—Synovial versus Subchondral Bone Pathology. Rheumatology.

[12] Mathiessen A., Conaghan P.G. (2017). Synovitis in Osteoarthritis: Current Understanding with Therapeutic Implications. Arthritis Res. Ther..

[13] Sanchez-Lopez E., Coras R., Torres A., Lane N.E., Guma M. (2022). Synovial Inflammation in Osteoarthritis Progression. Nat. Rev. Rheumatol..

[14] Thomson A., Hilkens C.M.U. (2021). Synovial Macrophages in Osteoarthritis: The Key to Understanding Pathogenesis?. Front. Immunol..

[15] Daghestani H.N., Pieper C.F., Kraus V.B. (2015). Soluble Macrophage Biomarkers Indicate Inflammatory Phenotypes in Patients With Knee Osteoarthritis. Arthritis Rheumatol..

[16] Bondeson J., Wainwright S.D., Lauder S., Amos N., Hughes C.E. (2006). The Role of Synovial Macrophages and Macrophage-Produced Cytokines in Driving Aggrecanases, Matrix Metalloproteinases, and Other Destructive and Inflammatory Responses in Osteoarthritis. Arthritis Res. Ther..

[17] Zhang H., Cai D., Bai X. (2020). Macrophages Regulate the Progression of Osteoarthritis. Osteoarthr. Cartil..

[18] Wu C.L., Harasymowicz N.S., Klimak M.A., Collins K.H., Guilak F. (2020). The Role of Macrophages in Osteoarthritis and Cartilage Repair. Osteoarthr. Cartil..

[19] Hunter D.J., Bierma-zeinstra S. (2019). Osteoarthritis. Lancet.

[20] Martel-Pelletier J., Barr A.J., Cicuttini F.M., Conaghan P.G., Cooper C., Goldring M.B., Goldring S.R., Jones G., Teichtahl A.J., Pelletier J.P. (2016). Osteoarthritis. Nat. Rev. Dis. Prim..

[21] Li Z., Huang Z., Bai L. (2021). Cell Interplay in Osteoarthritis. Front. Cell Dev. Biol..

[22] Chou C.H., Jain V., Gibson J., Attarian D.E., Haraden C.A., Yohn C.B., Laberge R.M., Gregory S., Kraus V.B. (2020). Synovial Cell Cross-Talk with Cartilage Plays a Major Role in the Pathogenesis of Osteoarthritis. Sci. Rep..

[23] Estell E.G., Silverstein A.M., Stefani R.M., Lee A.J., Murphy L.A., Shah R.P., Ateshian G.A., Hung C.T. (2019). Cartilage Wear Particles Induce an Inflammatory Response Similar to Cytokines in Human Fibroblast-Like Synoviocytes. J. Orthop. Res..

[24] Silverstein A.M., Stefani R.M., Sobczak E., Tong E.L., Attur M.G., Shah R.P., Bulinski J.C., Ateshian G.A., Hung C.T. (2017). Toward Understanding the Role of Cartilage Particulates in Synovial Inflammation. Osteoarthr. Cartil..

[25] Kraus V.B., McDaniel G., Huebner J.L., Stabler T.V., Pieper C.F., Shipes S.W., Petry N.A., Low P.S., Shen J., McNearney T.A. (2016). Direct in Vivo Evidence of Activated Macrophages in Human Osteoarthritis. Osteoarthr. Cartil..

[26] Beekhuizen M., Gierman L.M., van Spil W.E., Van Osch G.J.V.M., Huizinga T.W.J., Saris D.B.F., Creemers L.B., Zuurmond A.-M. (2013). An Explorative Study Comparing Levels of Soluble Mediators in Control and Osteoarthritic Synovial Fluid. Osteoarthr. Cartil..

[27] Molnar V., Matišić V., Kodvanj I., Bjelica R., Jeleč Ž., Hudetz D., Rod E., Čukelj F., Vrdoljak T., Vidović D. (2021). Cytokines and Chemokines Involved in Osteoarthritis Pathogenesis. Int. J. Mol. Sci..

[28] Monibi F., Roller B., Stoker A., Garner B., Bal S., Cook J. (2015). Identification of Synovial Fluid Biomarkers for Knee Osteoarthritis and Correlation with Radiographic Assessment. J. Knee Surg..

[29] Li L., Jiang B.-E. (2015). Serum and Synovial Fluid Chemokine Ligand 2/Monocyte Chemoattractant Protein 1 Concentrations Correlates with Symptomatic Severity in Patients with Knee Osteoarthritis. Ann. Clin. Biochem. Int. J. Lab. Med..

[30] Nair A., Gan J., Bush-Joseph C., Verma N., Tetreault M.W., Saha K., Margulis A., Fogg L., Scanzello C.R. (2015). Synovial Chemokine Expression and Relationship with Knee Symptoms in Patients with Meniscal Tears. Osteoarthr. Cartil..

[31] Deshmane S.L., Kremlev S., Amini S., Sawaya B.E. (2009). Monocyte Chemoattractant Protein-1 (MCP-1): An Overview. J. Interf. Cytokine Res..

[32] Raghu H., Lepus C.M., Wang Q., Wong H.H., Lingampalli N., Oliviero F., Punzi L., Giori N.J., Goodman S.B., Chu C.R. (2017). CCL2/CCR2, but Not CCL5/CCR5, Mediates Monocyte Recruitment, Inflammation and Cartilage Destruction in Osteoarthritis. Ann. Rheum. Dis..

[33] Agere S.A., Akhtar N., Watson J.M., Ahmed S. (2017). RANTES/CCL5 Induces Collagen Degradation by Activating MMP-1 and MMP-13 Expression in Human Rheumatoid Arthritis Synovial Fibroblasts. Front. Immunol..

[34] Russo R.C., Garcia C.C., Teixeira M.M., Amaral F.A. (2014). The CXCL8/IL-8 Chemokine Family and Its Receptors in Inflammatory Diseases. Expert Rev. Clin. Immunol..

[35] Islam S., Mir A.R., Abidi M., Talha M., Zafar A., Habib S., Moinuddin (2018). Methylglyoxal modified IgG generates autoimmune response in rheumatoid arthritis. Int. J. Biol. Macromol..

[36] Mehana E.-S.E., Khafaga A.F., El-Blehi S.S. (2019). The Role of Matrix Metalloproteinases in Osteoarthritis Pathogenesis: An Updated Review. Life Sci..

[37] Mariani E., Pulsatelli L., Facchini A. (2014). Signaling Pathways in Cartilage Repair. Int. J. Mol. Sci..

[38] Jenei-Lanzl Z., Meurer A., Zaucke F. (2019). Interleukin-1β Signaling in Osteoarthritis—Chondrocytes in Focus. Cell. Signal..

[39] Liao Y., Ren Y., Luo X., Mirando A.J., Long J.T., Leinroth A., Ji R.-R., Hilton M.J. (2022). Interleukin-6 Signaling Mediates Cartilage Degradation and Pain in Posttraumatic Osteoarthritis in a Sex-Specific Manner. Sci. Signal..

[40] Rose-John S. (2018). Interleukin-6 Family Cytokines. Cold Spring Harb. Perspect. Biol..

[41] Ripmeester E.G.J., Timur U.T., Caron M.M.J., Welting T.J.M. (2018). Recent Insights into the Contribution of the Changing Hypertrophic Chondrocyte Phenotype in the Development and Progression of Osteoarthritis. Front. Bioeng. Biotechnol..

[42] Wang M., Sampson E.R., Jin H., Li J., Ke Q.H., Im H.J., Chen D. (2013). MMP13 Is a Critical Target Gene during the Progression of Osteoarthritis. Arthritis Res. Ther..

[43] Gao Y., Liu S., Huang J., Guo W., Chen J., Zhang L., Zhao B., Peng J., Wang A., Wang Y. (2014). The ECM-Cell Interaction of Cartilage Extracellular Matrix on Chondrocytes. Biomed. Res. Int..

[44] Bauer C., Niculescu-Morzsa E., Jeyakumar V., Kern D., Späth S.S., Nehrer S. (2016). Chondroprotective effect of high-molecular-weight hyaluronic acid on osteoarthritic chondrocytes in a co-cultivation inflammation model with M1 macrophages. J. Inflamm..

[45] Mehta S., Akhtar S., Porter R.M., Önnerfjord P., Bajpayee A.G. (2019). Interleukin-1 receptor antagonist (IL-1Ra) is more effective in suppressing cytokine-induced catabolism in cartilage-synovium co-culture than in cartilage monoculture. Arthritis Res. Ther..

[46] Pagani S., Minguzzi M., Sicuro L., Veronesi F., Santi S., Scotto D’Abusco A., Fini M., Borzì R.M. (2019). The N-Acetyl Phenylalanine Glucosamine Derivative Attenuates the Inflammatory/Catabolic Environment in a Chondrocyte-Synoviocyte Co-Culture System. Sci. Rep..

[47] Muenzebrock K.A., Kersten V., Alblas J., Garcia J.P., Creemers L.B. (2022). The Added Value of the “Co” in Co-Culture Systems in Research on Osteoarthritis Pathology and Treatment Development. Front. Bioeng. Biotechnol..

[48] Ormandy E., Schuppli C. (2014). Public Attitudes toward Animal Research: A Review. Animals.

[49] Wang Q., Rozelle A.L., Lepus C.M., Scanzello C.R., Song J.J., Larsen D.M., Crish J.F., Bebek G., Ritter S.Y., Lindstrom T.M. (2011). Identification of a central role for complement in osteoarthritis. Nat Med..

