# Supplementary material for: Pararamosis, a Neglected Tropical Disease Induced by Premolis semirufa Caterpillar Toxins: Investigating Their Effects on Synovial Cell Inflammation
Source: Int J Mol Sci. 2024 Dec 6;25(23):13149. doi: 10.3390/ijms252313149 (PMC11641946; doi:10.3390/ijms252313149)
Supplement: Supplementary file 1 [file ijms-25-13149-s001.zip › ijms-3282394-supplementary/ijms-3282394-supplementary figures.pdf]

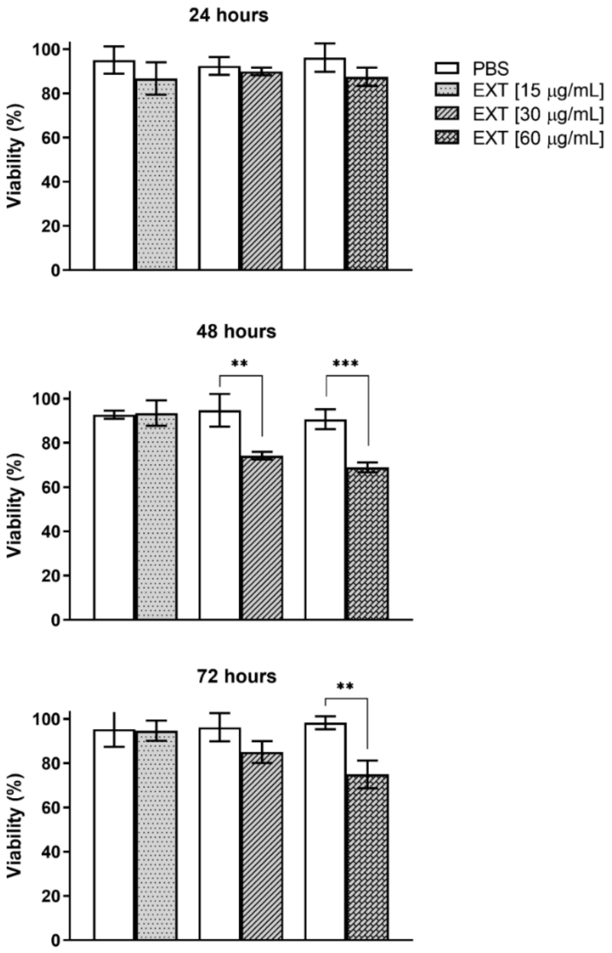

Figure S1. Synoviocyte viability. Synoviocytes were cultured in a 96-well plate at a concentration 1×10^4 cells/well and treated with increasing concentrations of Pararama hair extract (EXT 15, 30 or 60 μg/mL *per* well) for 24, 48 or 72 hours. Cell viability was assessed using the MTT assay. The data represent two experiments conducted in triplicate, and results are expressed as the mean of the triplicates ± standard error of the mean (SEM). Statistical analysis was performed using a t-test to compare each treatment condition against the negative control (PBS). Significance levels are indicated as $^{**}p \leq 0.01$ and $^{***}p \leq 0.001$.

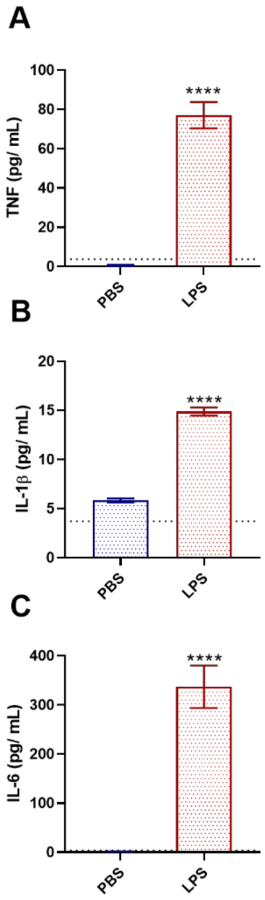

Figure S2. Cytokine levels in THP-1-derived macrophages treated with LPS. THP-1 cells (2.5×10^4 cells/well) were seeded in a 96-well transwell insert and differentiated into macrophages by treatment with 50 nM PMA for 48 hours, followed by a 24-hour rest period in PMA-free medium. The cells were then stimulated with LPS (100 ng/mL), and the culture supernatants were collected to assess cytokine concentrations using a cytometric bead array (CBA). Results are expressed as the mean concentrations of the cytokines ± standard error of the mean (SEM). Statistical analysis was performed using a t-test comparing each treatment group to the corresponding controls in PBS. Significance is indicated as ****$p \leq 0.0001$. The dotted line represents the limit of detection for each analyte.

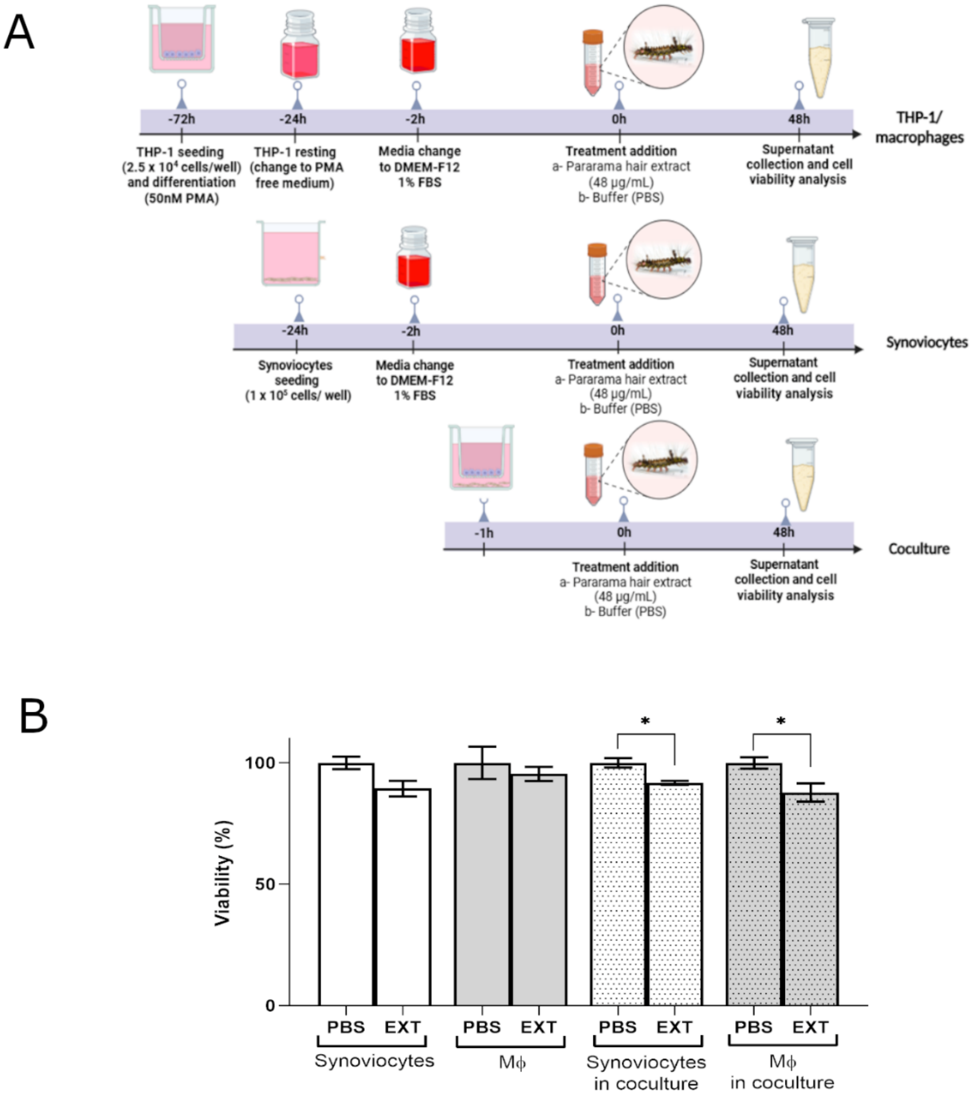

Figure S3. Workflow and treatment of synoviocyte and macrophage cocultures in a transwell system: experimental setup and viability assessment. THP-1 cells (2.5 × 104 cells/well) were seeded into 96-well transwell inserts with a pore size of 0.4 µm, where they were differentiated into macrophages by treatment with 50 nM PMA for 48 hours, followed by a 24-hour rest period in PMA-free medium. In parallel, synoviocytes (1 × 105 cells/well) were seeded in 24-well plates and cultured for 24 hours. Inserts containing the THP-1-derived macrophages (MΦ) were then transferred onto the top of the synoviocyte cultures. Some monocultures were maintained separately for comparative analysis. Subsequently, Pararama hair extract (EXT - 48 µg/mL) or PBS was added to the cell cultures. After 48 hours of incubation, supernatants were collected for protein concentration analysis, and the viability of mono- or cocultured cells was assessed. (A) The schematic representation was created using BioRender (https://biorender.com/). (B) Cell viability, expressed as a percentage, was measured using the MTT assay. The data were analyzed using a t-test comparing treated (EXT) versus each control group (PBS) within each cell population. Significance is indicated as *$p \leq 0.05$.

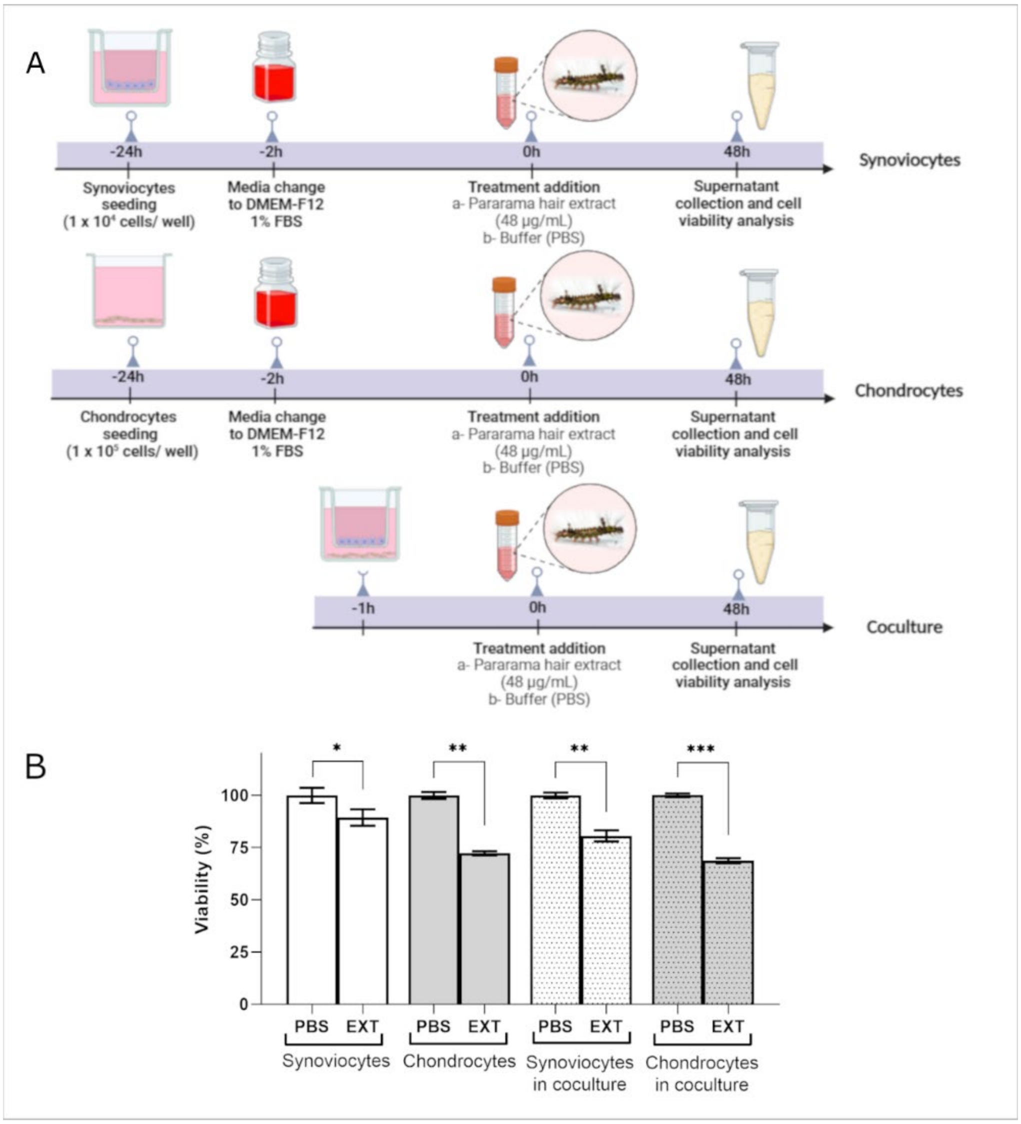

Figure S4. Workflow and treatment of synoviocyte and chondrocyte cocultures in a transwell system: experimental design and viability assessment. Synoviocytes (1×10^4 cells/well) were seeded into 96-well transwell inserts with a pore size of 0.4 µm, while chondrocytes (1×10^5 cells/well) were seeded in 24-well plates. The cells were cultured for 24 hours, after which the inserts containing the synoviocytes were transferred to the top of the chondrocyte cultures. Some monocultures were maintained separately for comparative analysis. Subsequently, Pararama hair extract (EXT- 48 µg/mL) or PBS was added to the cell cultures. After 48 hours of treatment, supernatants were collected for protein concentration analysis, and the viability of mono- or cocultured cells was assessed. (A) The schematic representation was created using BioRender (https://biorender.com/). (B) Cell viability, expressed as a percentage, was measured using the MTT assay. The data were analyzed using a t-test comparing treated (EXT) versus each control group (PBS) within each cell population. Significance levels are indicated as $*p \le 0.05$, $**p \le 0.01$, and $***p \le 0.001$.

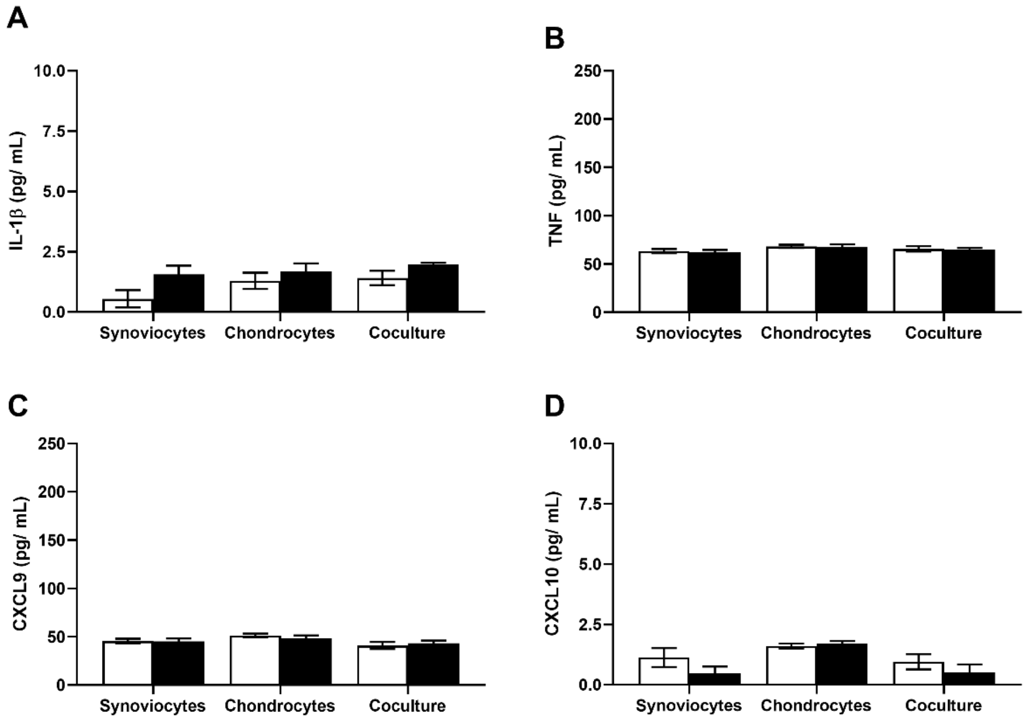

Supplementary Figure S5. Cytokine and chemokine levels in cocultured synoviocytes and chondrocytes induced by Pararama hair extract. Synoviocytes, chondrocytes, and cocultured cells in the transwell system were treated with Pararama hair extract (EXT; 48 μg/mL) or PBS (as a negative control) for 48 h. Supernatants of the cultures were collected to assess the concentrations of cytokines and chemokines via a cytometric bead array (CBA) (A-D). The results summarize two independent experiments, each performed in triplicate, and are expressed as the means of the concentrations of the molecules ± SEM. The data were analyzed using two-way ANOVA and Tukey's post hoc test. No significance was detected. Cytokine and chemokine levels in cocultured synoviocytes and chondrocytes induced by Pararama hair extract. Synoviocytes, chondrocytes, and cocultured cells in the transwell system were treated with Pararama hair extract (EXT; 48 μg/mL) or PBS (as a negative control) for 48 h. Supernatants of the cultures were collected to assess the concentrations of cytokines and chemokines via a cytometric bead array (CBA) (A-D). The results summarize two independent experiments, each performed in triplicate, and are expressed as the means of the concentrations of the molecules ± SEM. The data were analyzed using two-way ANOVA and Tukey's post hoc test. No significance was detected.
