# Supplementary material for: Pararamosis, a Neglected Tropical Disease Induced by Premolis semirufa Caterpillar Toxins: Investigating Their Effects on Synovial Cell Inflammation
Source: Int J Mol Sci. 2024 Dec 6;25(23):13149. doi: 10.3390/ijms252313149 (PMC11641946; doi:10.3390/ijms252313149)
Supplement: Supplementary file 1 [file ijms-25-13149-s001.zip › ijms-3282394-supplementary/ijms-3282394-supplementary table.pdf]

Supplementary Table S1. Key inflammatory factors in osteoarthritic pathogenesis [13,14,27,49] modulated in Pararamosis models.

Inflammatory factor	Modulation	Model
		Inflammatory cytokines
**IL-1β**	Levels upregulated in macrophage supernatant and unaltered in paw samples of mouse	Macrophages and cocultured macrophages and synoviocytes treated with Pararama hair extract [present report]; mouse inoculated with Pararama hair extract [6]
**IL-18**	RNA levels downregulated in chondrocytes	Human chondrocytes treated with Pararama hair extract [10]
**IL-6**	Levels upregulated in paw samples of mouse and supernatants of chondrocyte and synoviocyte cultures	Mouse inoculated with Pararama hair extract [6]; human chondrocytes treated with Pararama hair extract [10]; human synoviocytes, human chondrocytes and cocultured cells treated with Pararama hair extract [present report]
**TNF-α**	Levels upregulated in plasma, paw samples of mouse, and supernatants of macrophage cultures	Mouse inoculated with Pararama hair extract [6]; human whole blood model stimulated with Pararama hair extract [7]; Macrophages treated with Pararama hair extract [present report]
**IL-17**	Levels upregulated in plasma and paw samples of mouse	Mouse inoculated with Pararama hair extract [6]; human whole blood model stimulated with Pararama hair extract [7]
**IFN-γ**	Levels upregulated in paw samples of mouse	Mouse inoculated with Pararama hair extract [6]
**IL-15**	Not evaluated	
**IL-22**	Not evaluated	
		Anti-inflammatory cytokines
**IL-4**	Levels upregulated in paw samples of mouse	Mouse inoculated with Pararama hair extract [6]
**IL-10**	Levels upregulated in paw samples of mouse and not modulated in supernatants of synoviocytes and chondrocyte cultures	Mouse inoculated with Pararama hair extract [6]; human synoviocytes and chondrocytes treated with Pararama hair extract [present report]
**IL-13**	Not evaluated	
		Chemokines

**CXCL8 (IL-8)**	Levels upregulated in plasma and supernatants of macrophages, synoviocytes and chondrocytes cultures	Human whole blood model stimulated with Pararama hair extract [7]; human chondrocytes treated with Pararama hair extract [10]; macrophages, synoviocytes, chondrocytes and cocultured cells treated with Pararama hair extract [present report]
**CCL5 (RANTES)**	Levels upregulated in plasma and supernatants of macrophages, synoviocytes and chondrocytes cultures	Human whole blood model stimulated with Pararama hair extract [7]; macrophages, synoviocytes, chondrocytes and cocultured cells treated with Pararama hair extract [present report]
**CCL2 (MCP-10)**	Levels upregulated in plasma and supernatants of macrophages, synoviocytes and chondrocytes cultures	Human whole blood model stimulated with Pararama hair extract [7]; human chondrocytes treated with Pararama hair extract [10]; macrophages, synoviocytes, chondrocytes and cocultured cells treated with Pararama hair extract [present report]
**CXCL9 (MIG)**	Levels upregulated in plasma and supernatants of macrophages and synoviocytes cultures	Human whole blood model stimulated with Pararama hair extract [7]; macrophages, synoviocytes and cocultured cells treated with Pararama hair extract [present report]
**CXCL10 (IP-10)**	Levels upregulated in plasma and supernatants of macrophages and synoviocytes cultures	Human whole blood model stimulated with Pararama hair extract [7]; macrophages, synoviocytes and cocultured cells treated with Pararama hair extract [present report]
	Growth factors	
**TGF-β**	Not modulated in transcriptomic analysis	Human chondrocytes treated with Pararama hair extract [10]
**VEGF-α**	Upregulated in transcriptomic analysis	Human chondrocytes treated with Pararama hair extract [10]
	Extracellular matrix enzymes and inhibitors	
**MMP-1**	RNA levels upregulated in chondrocytes and in supernatants of synoviocytes and chondrocytes cultures	Human chondrocytes treated with Pararama hair extract [10]; synoviocytes, chondrocytes and cocultured cells treated with Pararama hair extract [present report]
**MMP-2**	Levels upregulated in supernatants of synoviocytes and chondrocytes cultures	Human chondrocytes treated with Pararama hair extract [10]; synoviocytes treated with Pararama hair extract [present report]
**MMP-3**	RNA levels upregulated in chondrocytes and in supernatants of synoviocytes and chondrocytes cultures	Human chondrocytes treated with Pararama hair extract [10]; synoviocytes, chondrocytes and cocultured cells treated with Pararama hair extract [present report]
**MMP-9**	Levels not modulated in cell supernatants	Human chondrocytes treated with Pararama hair extract [10]; synoviocytes treated with Pararama hair extract [present report]

**MMP-13**	RNA levels upregulated in chondrocytes and in supernatants of synoviocyte and chondrocyte cultures	Human chondrocytes treated with Pararama hair extract [10]; synoviocytes, chondrocytes and cocultured cells treated with Pararama hair extract [present report]
**ADAMTS4**	Levels not modulated in supernatants of chondrocyte cultures	Human chondrocytes treated with Pararama hair extract [10]
**ADAMTS5**	Not evaluated	
**TIMP-1**	Levels not modulated in supernatants of chondrocyte cultures	Human chondrocytes treated with Pararama hair extract [10]
	Complement system factors and Anaphylatoxins	
**C3**	Levels upregulated in supernatants of chondrocyte cultures	Human chondrocytes treated with Pararama hair extract [10]
**C4**	Levels upregulated in supernatants of chondrocyte cultures	Human chondrocytes treated with Pararama hair extract [10]
**C5**	Levels upregulated in supernatants of chondrocyte cultures	Human chondrocytes treated with Pararama hair extract [10]
**C3a**	Levels upregulated in plasma	Human whole blood model stimulated with Pararama hair extract [7]
**C4a**	Levels upregulated in plasma	Human whole blood stimulated with Pararama hair extract [7]
**C5a**	Levels upregulated in plasma	Human whole blood stimulated with Pararama hair extract [7]
**C5b-9**	Levels upregulated in plasma	Human whole blood stimulated with Pararama hair extract [7]
	Others	
**PGE-2**	Levels upregulated in supernatants of chondrocyte cultures	Human chondrocytes treated with Pararama hair extract [10]
**COX-2**	Upregulated in transcriptomic analysis	Human chondrocytes treated with Pararama hair extract [10]
**NO**	Not evaluated	
